# Supplementary material for: Annexin A5 as an immune checkpoint inhibitor and tumor-homing molecule for cancer treatment
Source: Nat Commun. 2020 Feb 28;11:1137. doi: 10.1038/s41467-020-14821-z (PMC7048819; doi:10.1038/s41467-020-14821-z)
Supplement: Supplementary file 1 — Supplemental Figures [file 41467_2020_14821_MOESM1_ESM.pdf]

Supplementary Information

Annexin A5 as an immune checkpoint inhibitor and tumor-bearing molecule for cancer treatment

Kang et al.

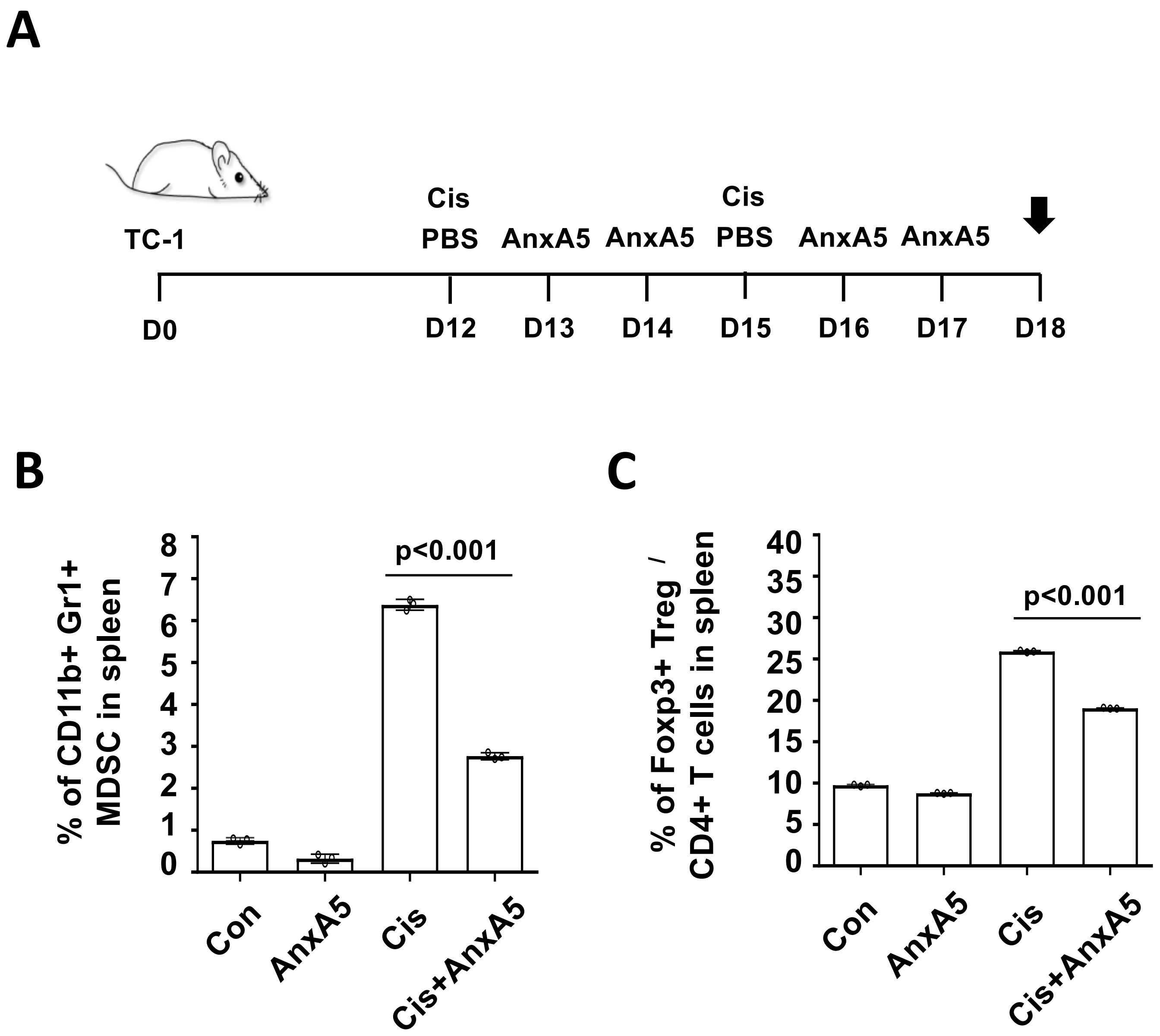

Supplementary Figure 1. Decrease of immune suppressive cells in the spleens after Annexin A5 treatment. C57BL/6 mice were injected with $2x10^5$ TC-1 cells/mouse subcutaneously on day 0. Mice were then treated intraperitoneally with 5mg/kg Cisplatin on days 12 and 15, and/or intravenously with 200µg/mice of Annexin A5 proteins on days 13, 14, 16, and 17. PBS was used as control. On day 18, spleens of mice were harvested. (A) Schematic diagram. (B-C) Bar graphs depicting the presence of CD11b+ GR1+ MDSCs (B) or CD4+ CD25+ Foxp3+ Treg cells (C) in the spleen following flow cytometry analysis. The error bars indicate mean ± SD. P-values were analyzed by Student's t-test (n=3). The results are representative of one of three independent experiments. Source data are provided as a Source Data file.

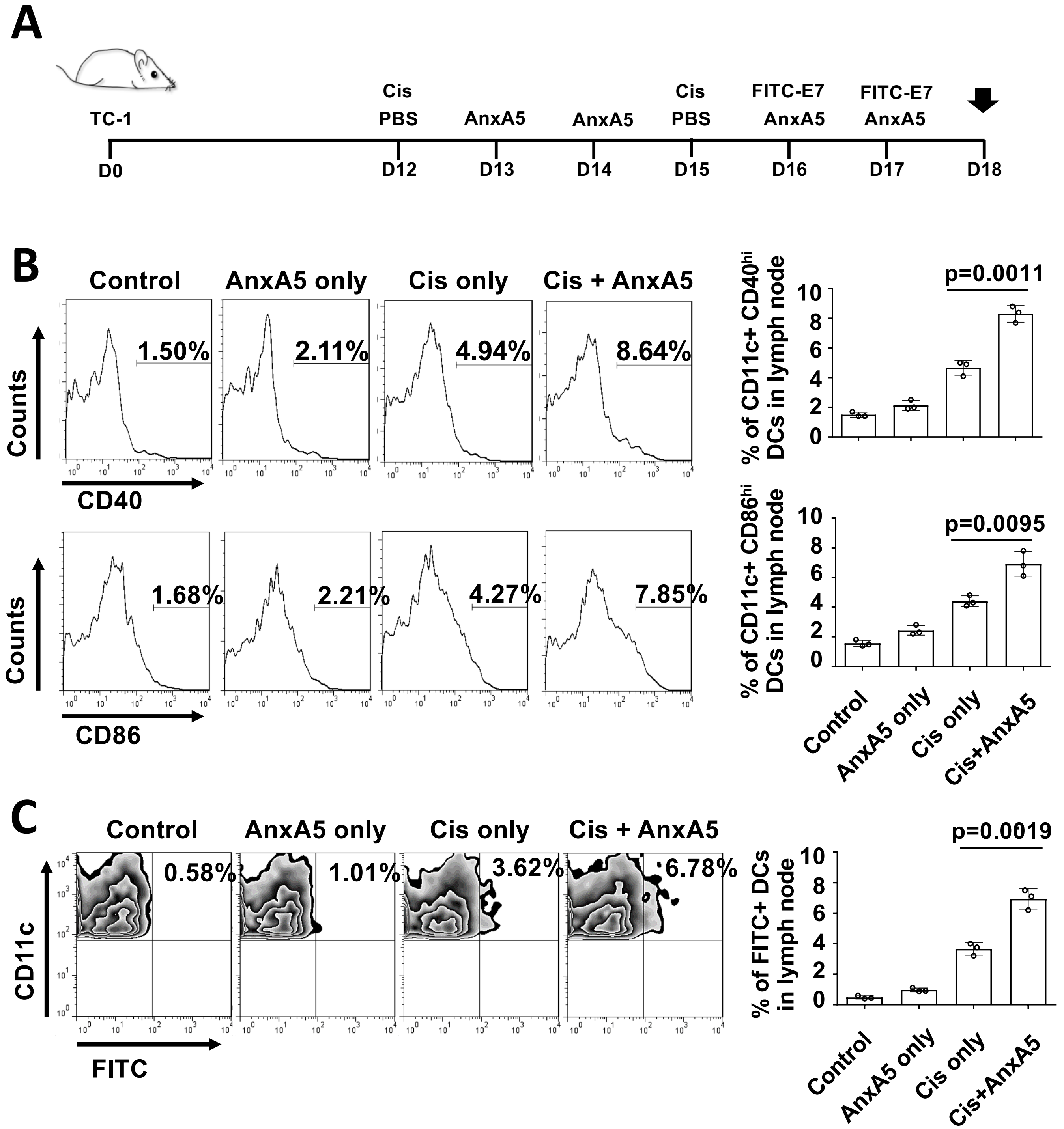

Supplementary Figure 2. Annexin A5 treatment increase dendritic cell maturation. C57BL/6 mice were injected with $2x10^5$ TC-1 cells/mouse subcutaneously on day 0. Mice were then treated intraperitoneally with 5mg/kg Cisplatin on days 12 and 15, and/or intravenously with 200µg/mice of Annexin A5 proteins on days 13, 14, 16, and 17. PBS was used as control. On day 18, draining LN DCs of mice were harvested. **(A)** Schematic diagram. **(B)** Representative flow cytometry analysis and bar graphs depicting the % of CD11c+ CD40+ or CD86+ DCs in the draining lymph nodes (n=3). (**C**) To assess the migration of CD11c+ dendritic cells to draining lymph nodes, TC-1 tumor bearing, cisplatin and/or AnxA5 treated mice were injected intratumorally with FITC-labeled E7 peptide as indicated. On day 18, DCs from the draining lymph node of mice were harvested. Figure showing representative flow cytometry analysis and bar graphs depicting the % of CD11c+ FITC+ DCs in the draining lymph node (n=3). The error bars indicate mean ± SD.P-values were analyzed by Student's t-test (n=3). The results are representative of one of three independent experiments. Source data are provided as a Source Data file.

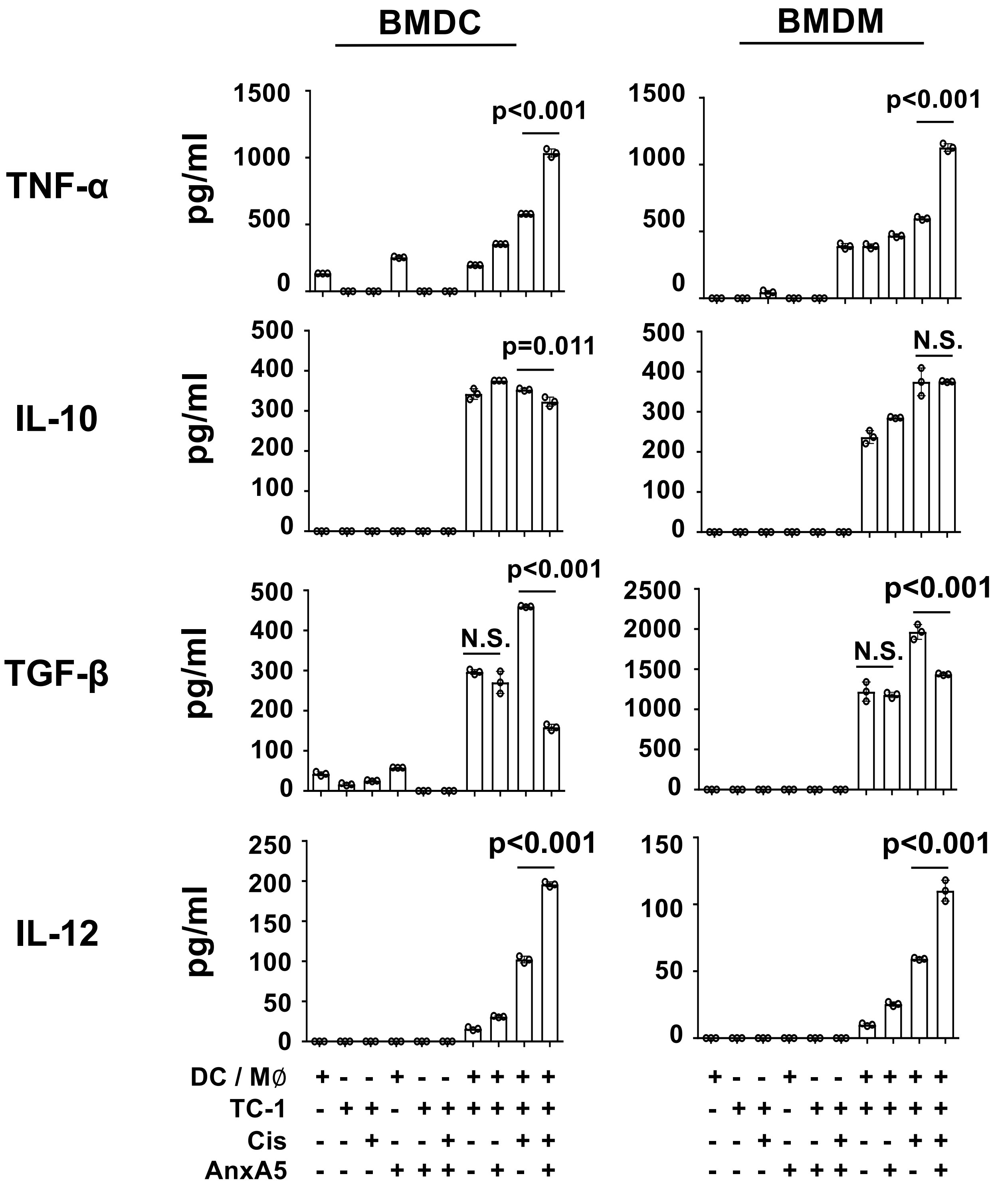

Supplementary Figure 3. Change of inflammatory cytokines after Annexin A5 treatment. 1x10^5 of TC-1 tumor cells were treated with or without 20μg/ml of cisplatin for 6 hours. The tumor cells were then washed twice with PBS and co-cultured with 1x10^5 of bone marrow derived dendritic cell or bone marrow derived macrophage with or without concomitant incubation with 20μg/ml Annexin A5 protein. 24hours after co-culturing, supernatants were collected and assessed for TNF-α, IL-10, TGF-β, and IL-12 cytokine levels by ELISA**.** Bar graph depicting the levels of cytokines in various treatment group (n=3). The error bars indicate mean ± SD. P-values were analyzed by Student's t-test (n=3). The results are representative of one of three independent experiments. Source data are provided as a Source Data file. N.S. = not significant.

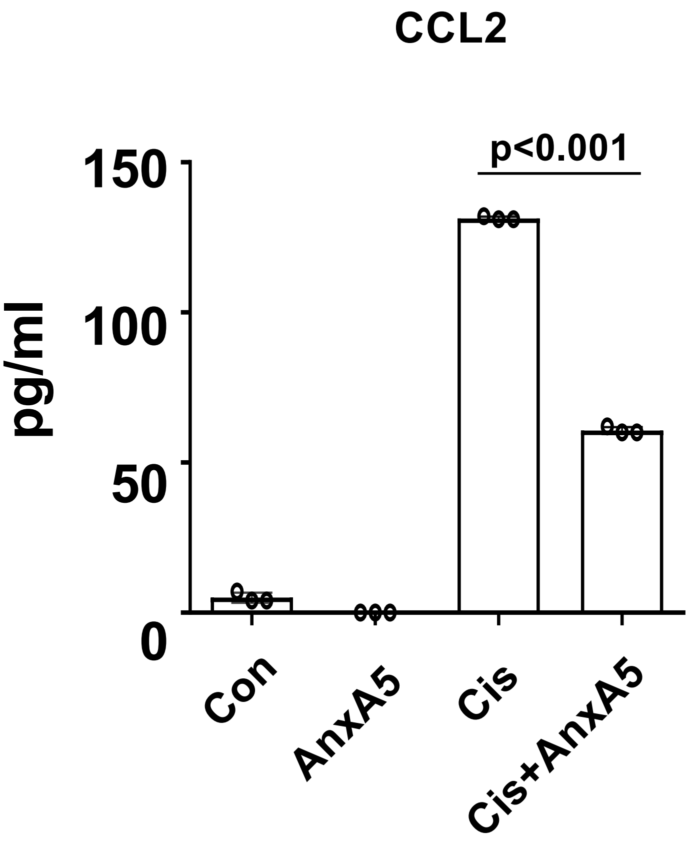

Supplementary Figure 4. Characterization of CCL2 chemokine after Annexin A5 treatment. C57BL/6 mice were injected with $2x10^5$ TC-1 cells/mouse subcutaneously on day 0. Mice were then treated intraperitoneally with 5mg/kg Cisplatin on days 12 and 15, and/or intravenously with 200µg/mice of Annexin A5 proteins on days 13, 14, 16, and 17. PBS was used as control. On day 18, tumor tissues were harvested, and the level of CCL2 chemokine in the tumor tissue of mice were measured by ELISA. The error bars indicate mean ± SD. P-values were analyzed by Student's t-test (n=3). The results are representative of one of three independent experiments. Source data are provided as a Source Data file.

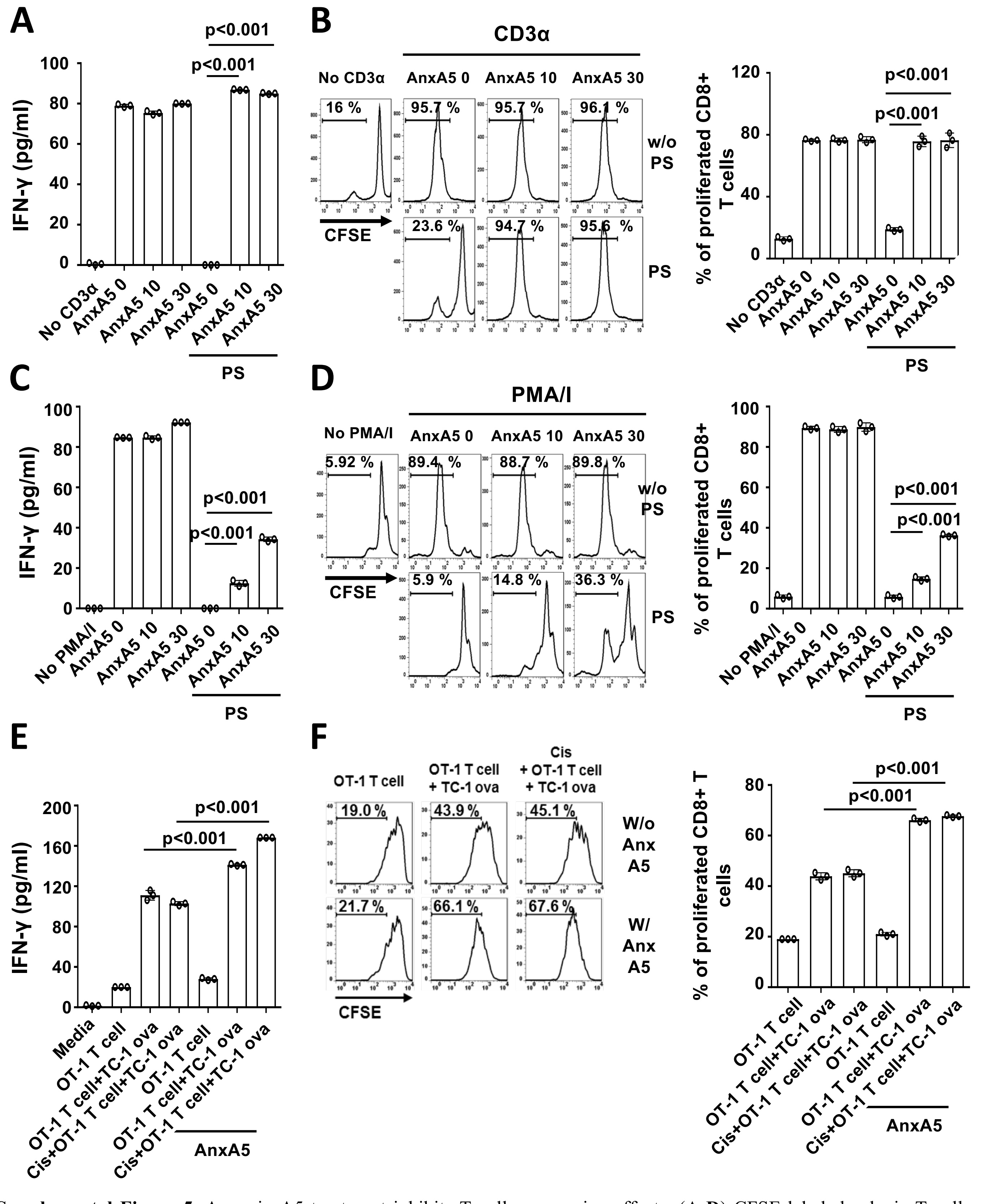

Supplemental Figure 5. Annexin A5 treatment inhibits T cell suppressive effects. (**A-D**) CFSE-labeled splenic T cells were stimulated for 18h with anti-CD3α mAb (10μg/ml) (**A-B**) or PMA (50ng/ml) plus Ionomycin (500ng/ml) (**C-D**) in the presence of Phosphatidyl-serine (5μg/ml) with or without co-treatment of Annexin A5 protein (10μg/ml or 30μg/ml). **(E-F)** $1x10^6$ CFSE labelled OVA specific CD8+ T cells were co-cultured with OVA expressing TC-1 cells pre-treated with or without cisplatin and/or 20μg/ml of Annexin A5 protein. (**A, C and E**) 24 hrs after incubation, the supernatants were collected and measured for IFN-γ levels using ELISA. Figure displaying bar graph of supernatant IFN-γ levels. (**B, D, and F**) Division of T cells were assessed by flow cytometry analysis based on CFSE dilution at 3 days after incubation. Figure displaying representative flow cytometry images and bar graphs of CD8+ CFSE+ T cell proliferation. The error bars indicate mean ± SD. For **A-F**, P-values were analyzed by Student's t-test (each group, n=3). The results are representative of one of three independent experiments. Source data are provided as a Source Data file.

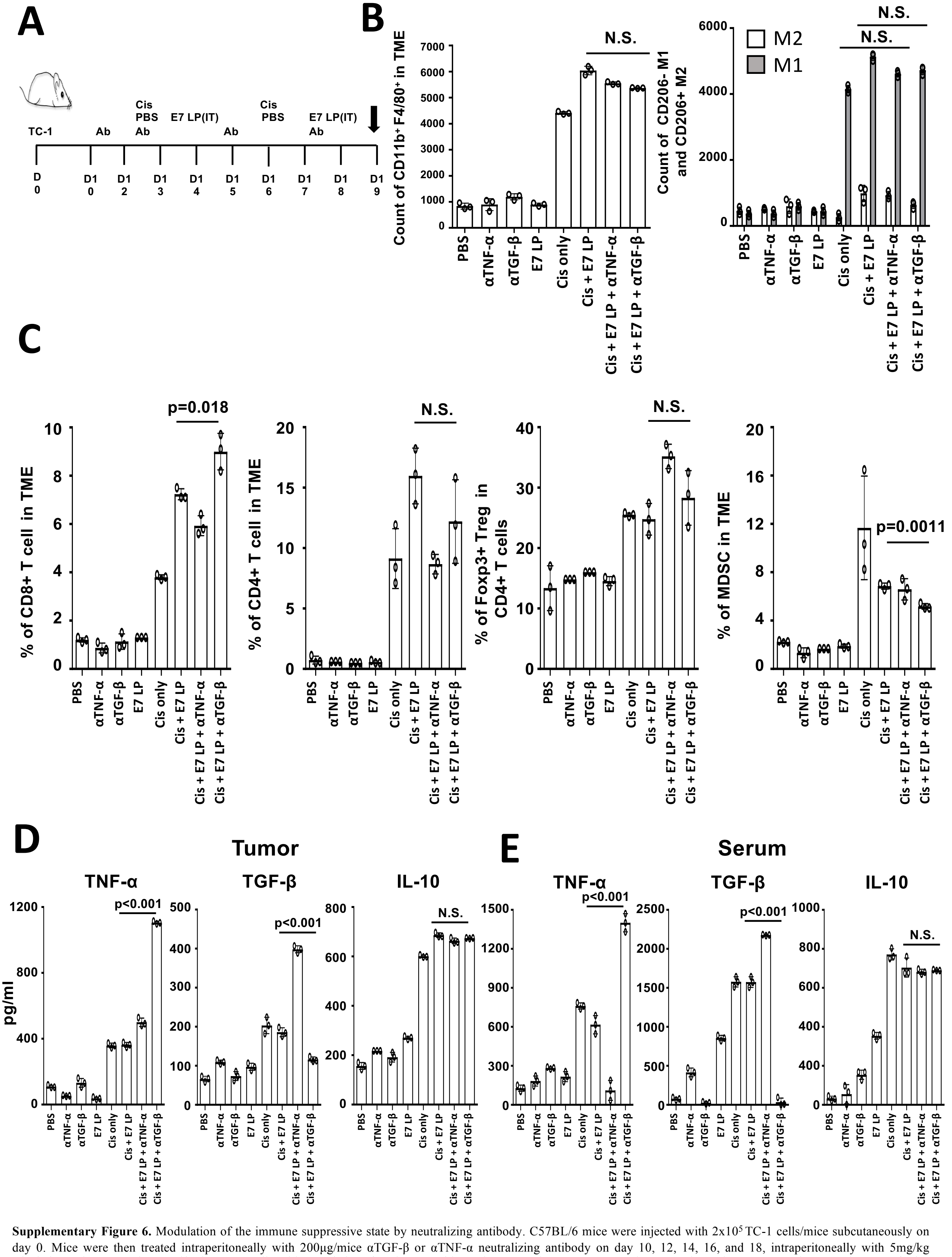

Supplementary Figure 6. Modulation of the immune suppressive state by neutralizing antibody. C57BL/6 mice were injected with $2x10^5$ TC-1 cells/mice subcutaneously on day 0. Mice were then treated intraperitoneally with 200μg/mice αTGF-β or αTNF-α neutralizing antibody on day 10, 12, 14, 16, and 18, intraperitoneally with 5mg/kg Cisplatin on days 12 and 15, and/or intratumorally with 20μg/mice of E7 long peptide on days 13 and 16. PBS was used as control. On day 19, tumor tissues and serum of mice were harvested. **(A)** Schematic diagram. **(B)** Bar graphs depicting the abundance of CD11b+ F4/80+ macrophages and their M1 / M2 distributions in the tumor tissue following flow cytometry analysis (n=3). **(C)** Bar graphs depicting the presence of CD8+ T cells, CD4+ T cells, Treg cells, and MDSCs in the tumor tissue following flow cytometry analysis (n=3). **(D-E)** Bar graphs depicting the levels of TNF-α, TGF-β, and IL-10 cytokines in the tumor tissue **(D)** and serum **(E)** of mice as measured by ELISA (n=3). The error bars indicate mean ± SD. For **A-E**, P-values were analyzed by Student's t-test (each group, n=3). N.S. = not significant. The results are representative of one of three independent experiments. Source data are provided as a Source Data file.

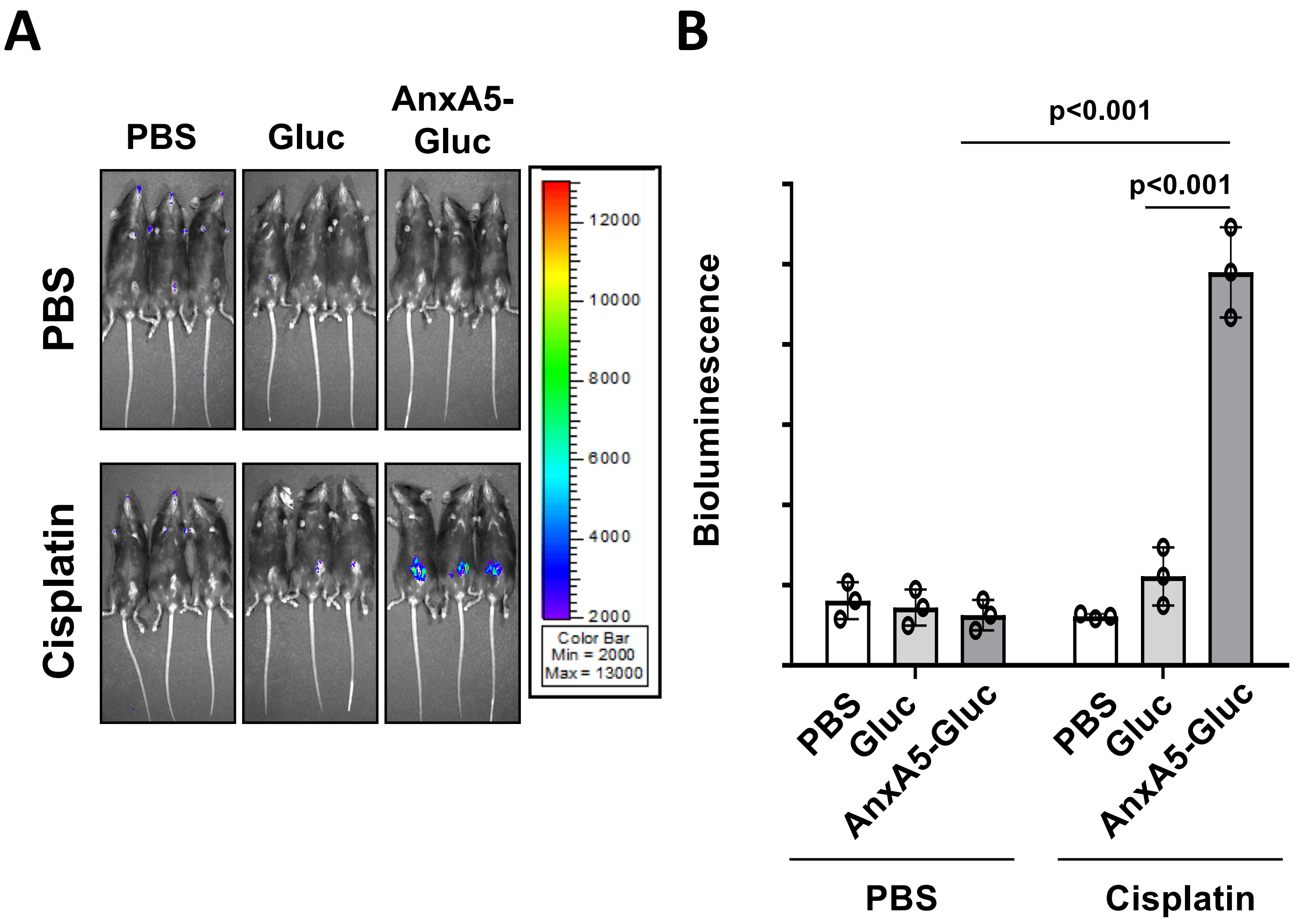

Supplementary Figure 7. Homing of Annexin A5 protein to tumor loci following cisplatin treatment. C57BL/6 mice were injected with $2x10^5$ TC-1 cells/mouse subcutaneously. 12 days later, tumor bearing mice were treated with or without 5mg/kg of cisplatin intra-peritoneally. After 2 days, 200µg/mice of PBS, Annexin A5 only, or Annexin A5-Gluc proteins were injected intravenously into the lateral tail vein and the bioluminescence was imaged one day later. **(A)** Representative bioluminescence imaging used to characterize the accumulation of Annexin A5 containing protein into tumor loci in tumor-bearing mice after cisplatin treatment. **(B)** Bar graph depicting the fluorescence intensity in tumor-bearing mice treated with the various groups (n=3). The error bars indicate mean ± SD. P-values were analyzed by Student's t-test (each group, n=3). The results are representative of one of three independent experiments. Source data are provided as a Source Data file.

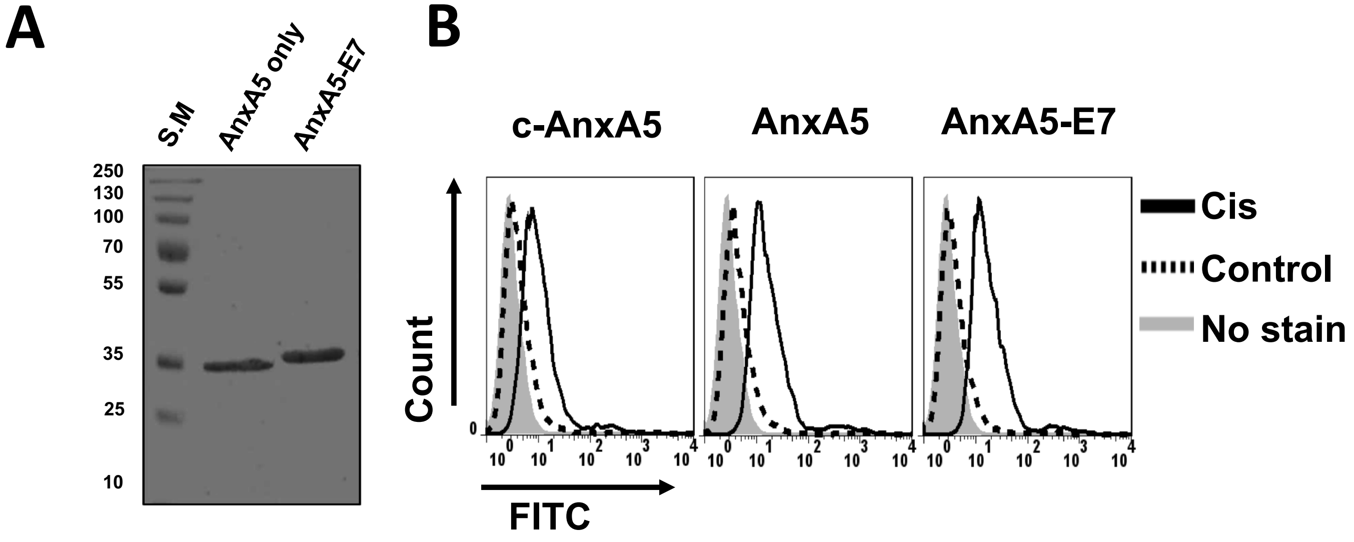

Supplementary Figure 8. Characterization of recombinant Annexin A5-E7 fusion protein. **(A)** Size and purity of recombinant Annexin A5 protein or Annexin A5-E7 fusion protein were assessed using 12% gradient SDS-PAGE followed by Coomassie brilliant blue staining in the same gel. **(B)** Functionality assessment of Annexin A5-E7 recombinant protein. $2x10^5$ TC-1 tumor cells treated with or without Cisplatin were incubated with commercially available FITC-Annexin A5, FITC-labeled recombinant Annexin A5 protein, or FITC-labeled recombinant Annexin A5-E7 fusion protein, followed by flow cytometry analysis. Source data are provided as a Source Data file.

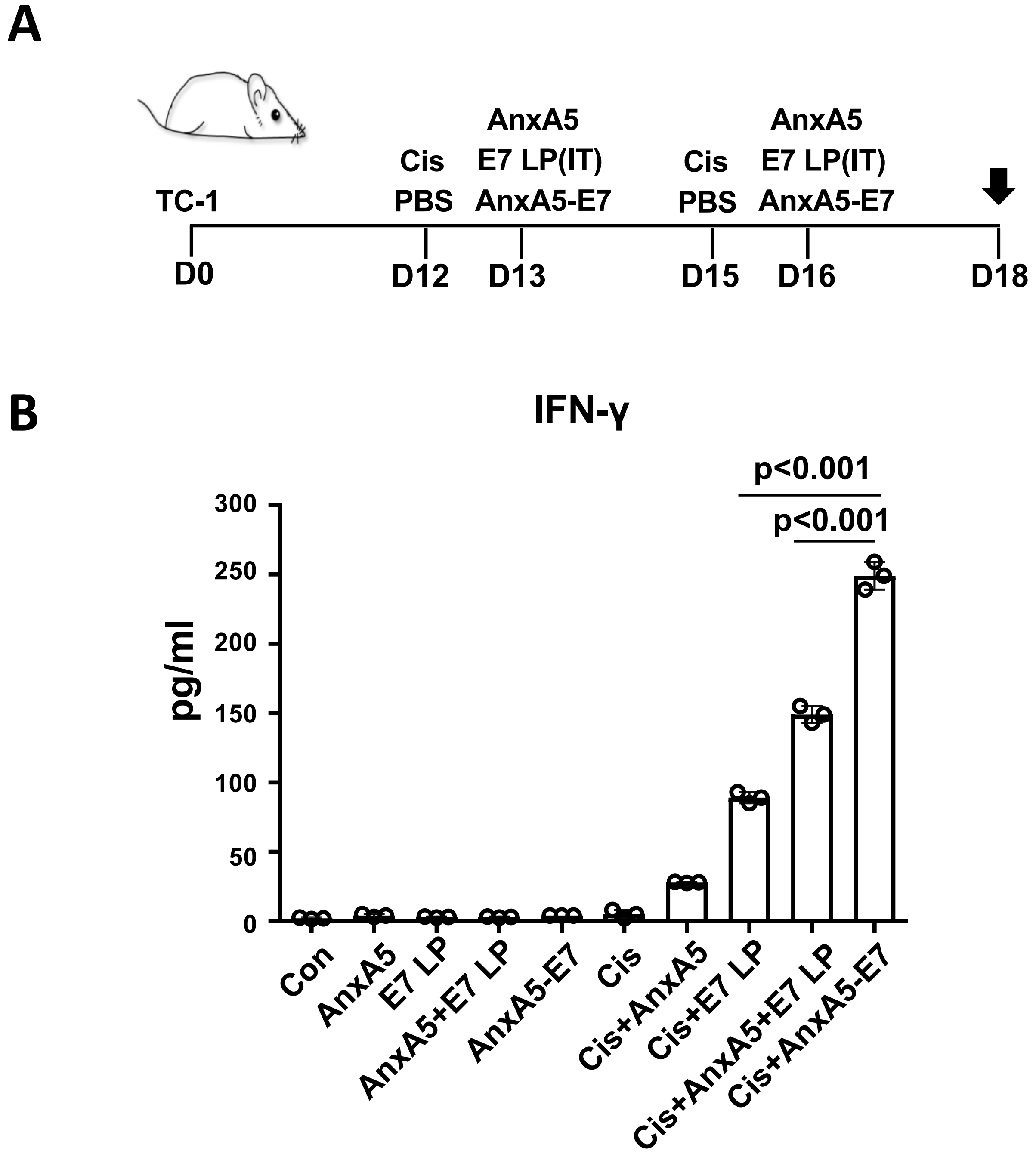

Supplementary Figure 9. Annexin A5 fusion protein enhances antigen presentation by dendritic cells. C57BL/6 mice were injected with $2x10^5$ TC-1 cells/mouse subcutaneously on day 0. Mice were then treated intraperitoneally with 5mg/kg Cisplatin on days 12 and 15, as well as 200μg/mice of AnxA5 intravenously, 200μg/mice of AnxA5-E7 intravenously, and/or 20μg/mice of E7 long peptide intratumorally on days 13 and 16. PBS was used as control. On day 18, tumor draining lymph nodes were harvested and isolated for CD11c+ dendritic cells. **(A)** Schematic diagram. **(B)** The isolated CD11c+ dendritic cells ($1x10^5$) were incubated with $1x10^6$ of CD8+ T cells harvested from AnxA5-E7 treated mice for 16h. Supernatants were collected and assessed for IFN-γ cytokine levels by ELISA**.** Bar graph depicting the levels of cytokines in various treatment group (n=3). The error bars indicate mean ± SD. P-values were analyzed by Student's t-test. The results are representative of one of three independent experiments. Source data are provided as a Source Data file.

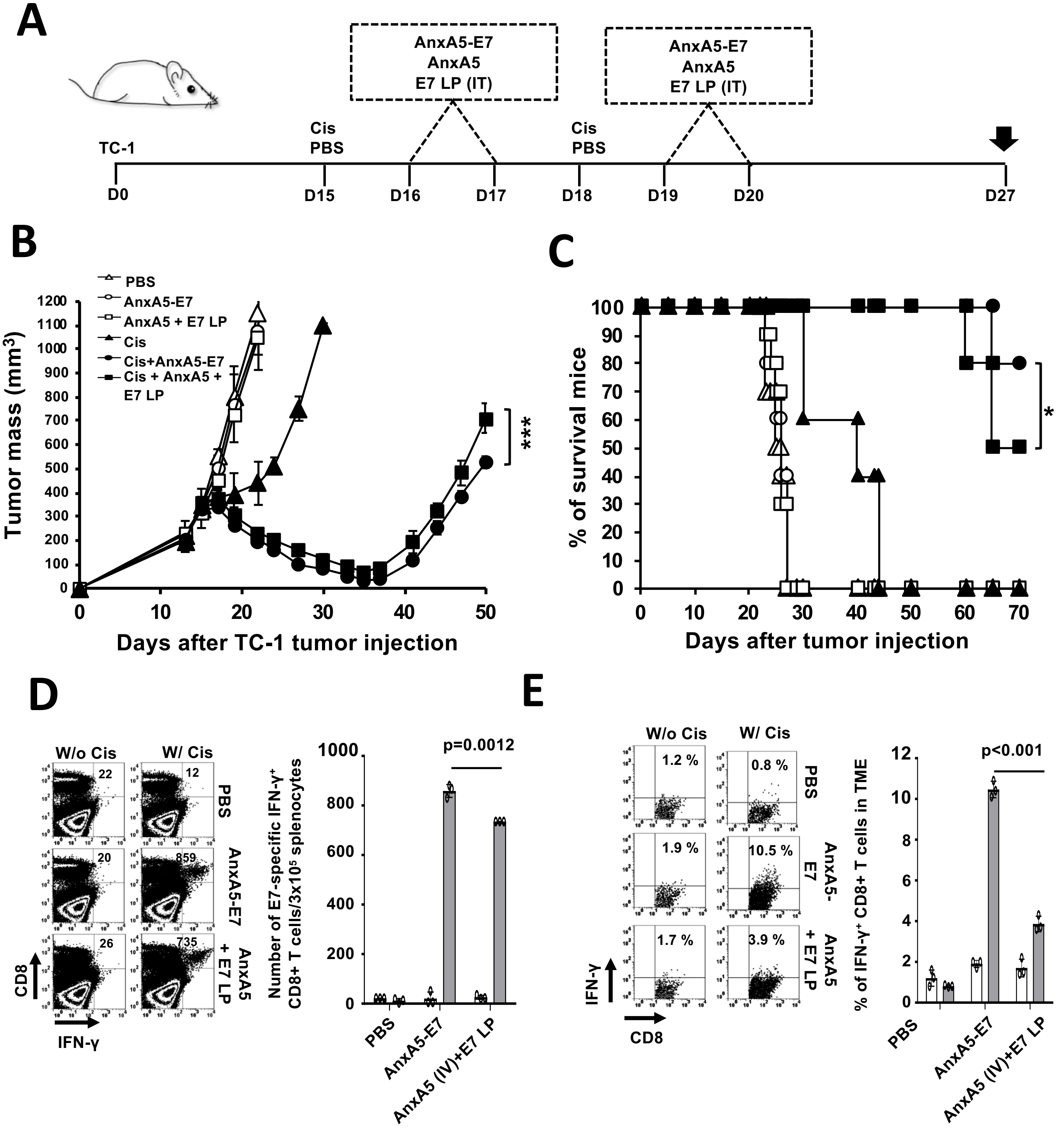

Supplementary Figure 10. Comparison of therapeutic antitumor effects by Annexin A5-E7 versus AnxA5 plus E7 peptide. C57BL/6 mice were injected with 2x10^5 TC-1 cells/mouse subcutaneously on day 0. On days 15 and 18, mice were treated intraperitoneally with 5mg/kg cisplatin. On days 16, 17, 19, and 20, mice were treated with 200μg/mice of AnxA5-E7 intravenously, 200μg/mice of AnxA5 intravenously, and/or 3.5μg/mice of E7 peptide intratumorally. PBS was used as control. **(A)** Schematic diagram. **(B)** Line graph depicts TC-1 tumor growth in different treatment groups over time (n=10)**.** P-values were determined by one-way ANOVA and Turkey's test. **(C)** Kaplan–Meier survival analysis of TC-1 tumor-bearing mice in different treatment groups and the overall P-value was calculated by the log-rank test (n=10). **(D-E)** One week after the last vaccination, spleens and tumors of mice in different treatment groups were harvested and analyzed for CD8+IFN-γ+ T cells by flow cytometry analysis. **(D)** Representative flow cytometry analysis and bar graph depicting the abundance of CD8+IFN-γ+ T cells in splenocytes of TC-1 tumor bearing mice in different treatment groups (n=3). **(E)** Representative flow cytometry analysis and bar graph depicting the abundance of CD8+IFN-γ+ tumor-infiltrating T cells in TC-1 tumor bearing mice in different treatment groups (n=3). The error bars indicate mean ± SD. For **D** and **E**, P-values were analyzed by Student's t-test. *P < 0.05, **P<0.01, ***P <0.001. The results are representative of one of three independent experiments. Source data are provided as a Source Data file.

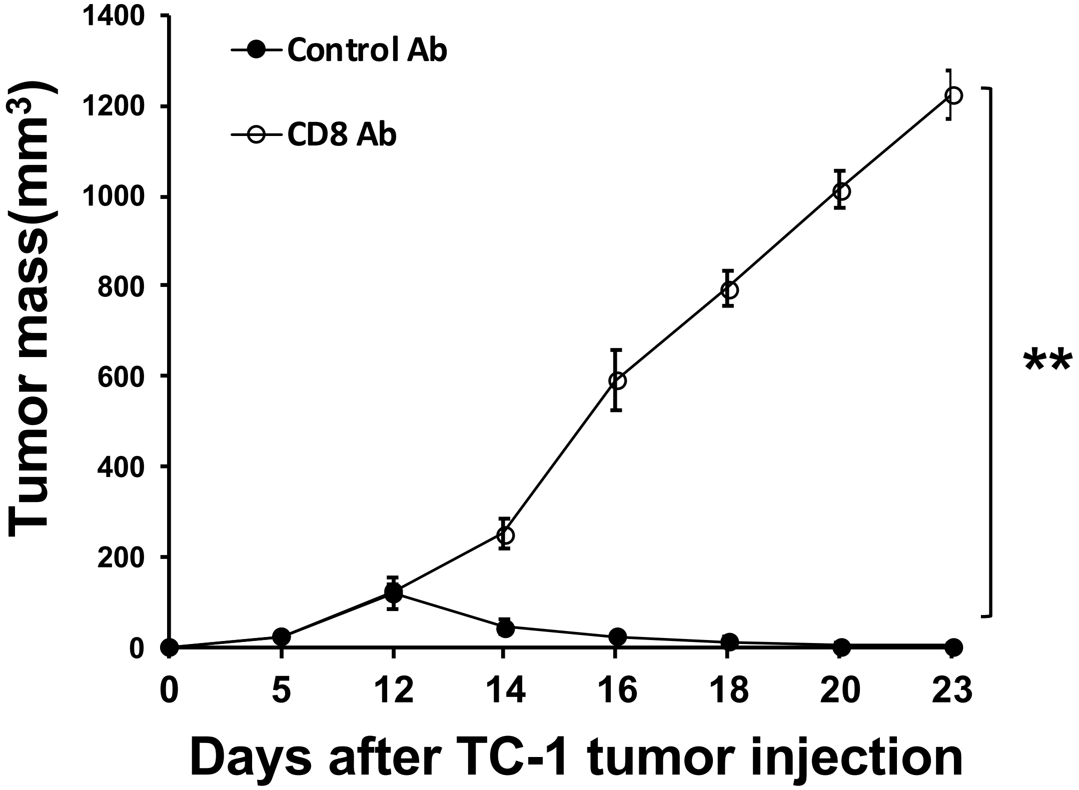

Supplementary Figure 11. Therapeutic antitumor effect by Annexin A5-E7 administration. C57BL/6 mice were challenged with TC-1 tumor cells and treated with cisplatin and AnxA5-E7 using the same dosages and schedules as described in Figure 5. 200μg/mice of anti-CD8 depleting antibodies or control IgG antibodies were administered intraperitoneally daily from day 12 to day 20. Line graph depicts TC-1 tumor growth in control group and CD8 depleted group over time (n=10). P-values were determined by one-way ANOVA and Turkey's test. The error bars indicate mean ± SD. *P < 0.05, **P<0.01, ***P <0.001. Source data are provided as a Source Data file.

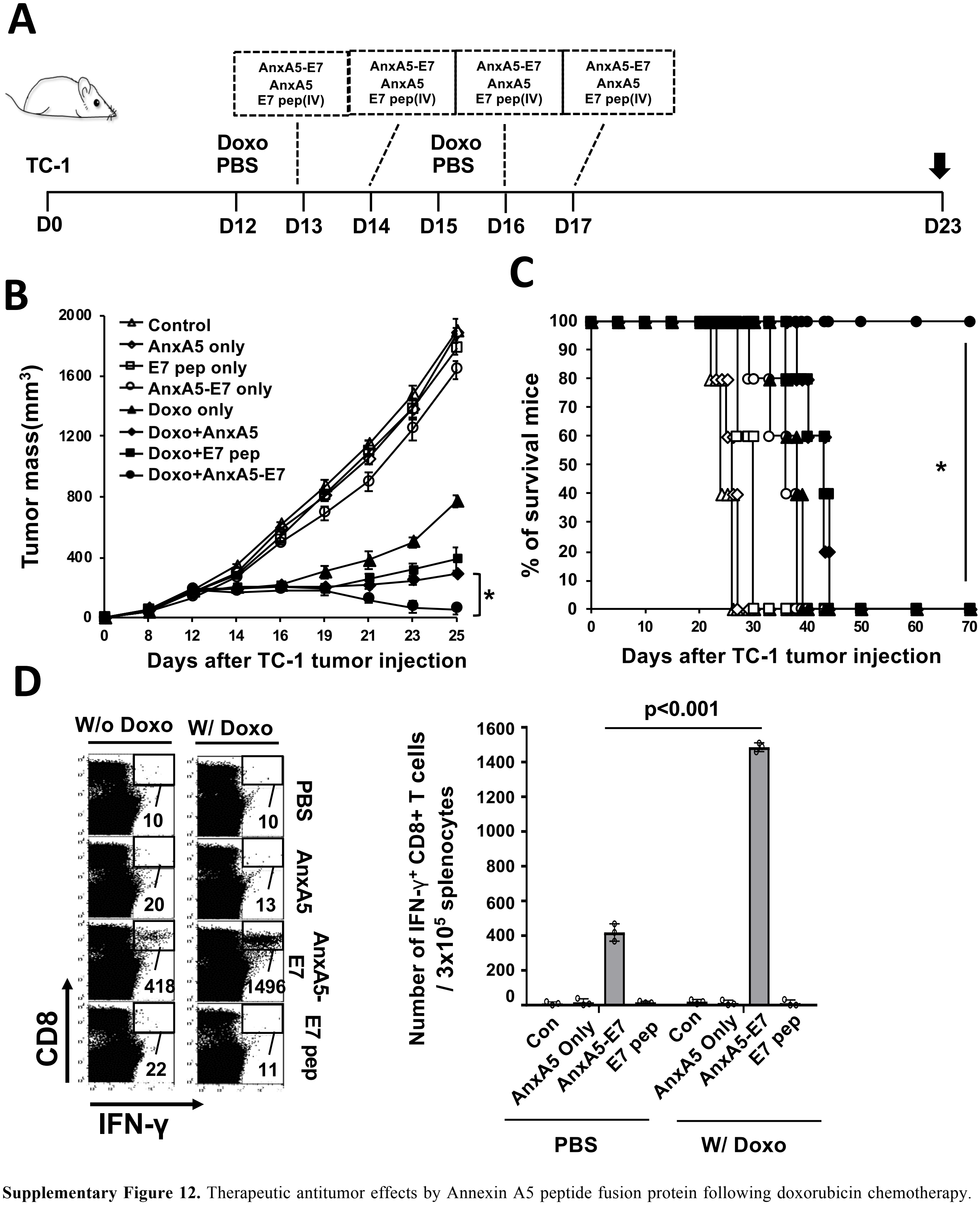

Supplementary Figure 12. Therapeutic antitumor effects by Annexin A5 peptide fusion protein following doxorubicin chemotherapy. C57BL/6 mice were injected with $2x10^5$ TC-1 cells/mouse subcutaneously on day 0. Mice were then treated intraperitoneally with 10mg/kg doxorubicin on days 12 and 15 and intravenously with 200µg/mice of AnxA5, 200µg/mice of AnxA5-E7, and/or 3.5µg/mice of E7 peptide on days 13, 14, 16, and 17. PBS was used as control. **(A)** Schematic diagram. **(B)** Line graph depicts TC-1 tumor growth in different treatment groups over time (n=10). P-values were determined by one-way ANOVA and Turkey's test. **(C)** Kaplan–Meier survival analysis of TC-1 tumor-bearing mice in different treatment groups (n=10), and the overall P-value was calculated by the log-rank test. **(D)** One week after the last vaccination, spleens of TC-1 tumor-bearing mice in different treatment groups were harvested and analyzed for CD8+IFN-γ+ T cells by flow cytometry analysis. Figure showing the representative flow cytometry analysis and bar graph depicting the abundance of CD8+IFN-γ+ T cells in splenocytes of TC-1 tumor bearing mice in different treatment groups (n=3). The error bars indicate mean ± SD. For **D**, P-values were analyzed by Student's t-test. *P < 0.05, **P<0.01, ***P <0.001, N.S. = not significant. The results are representative of one of three independent experiments. Source data are provided as a Source Data file.

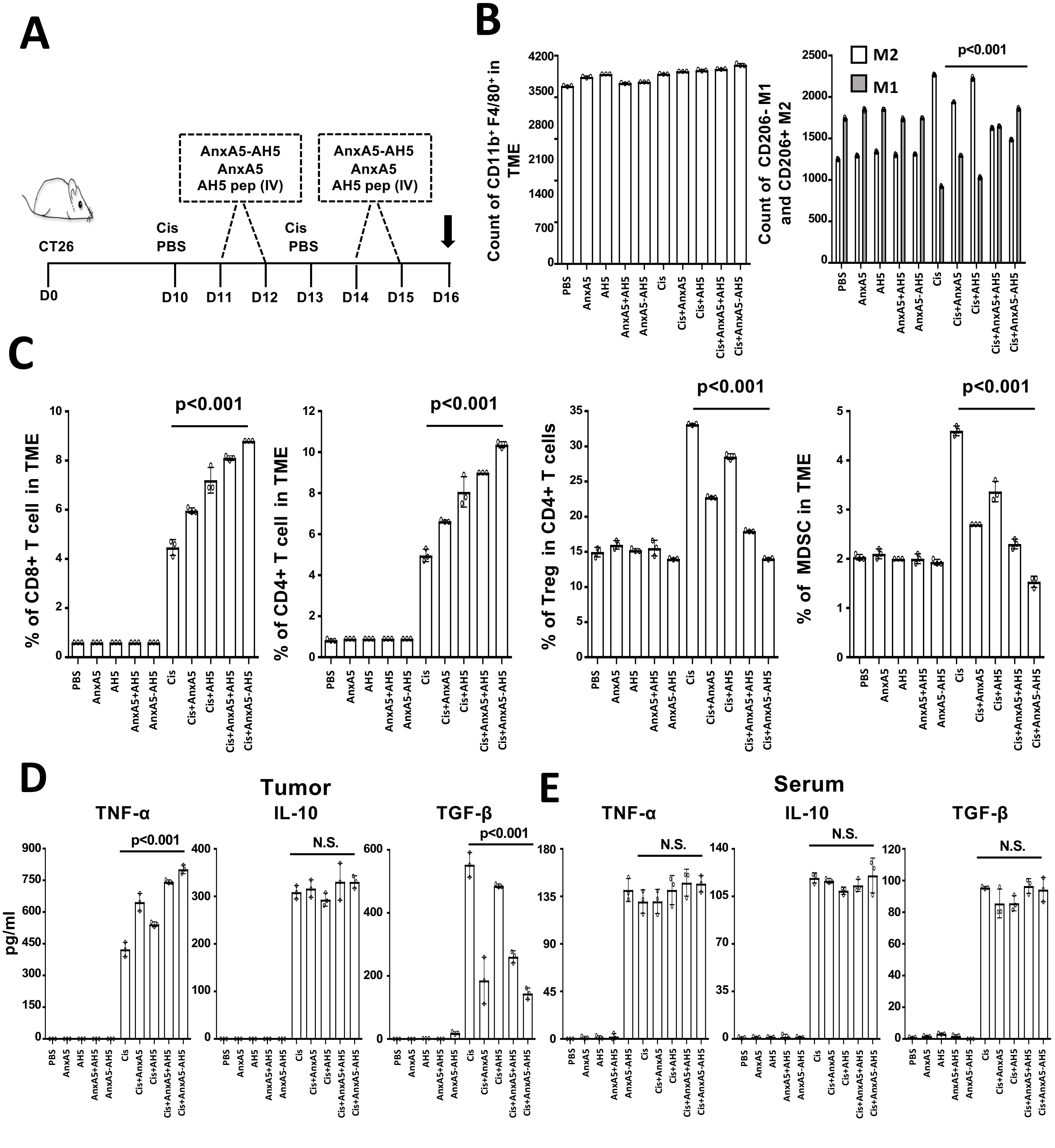

Supplementary Figure 13. Characterization of TME following Annexin A5 fusion protein treatment in CT-26 tumor bearing mice. BALB/c mice were injected with 5x10^5 CT-26 cells/mouse subcutaneously on day 0. Mice were then treated intraperitoneally with 5mg/kg Cisplatin on days 10 and 13, as well as intravenously with 200μg/mice of AnxA5, 200μg/mice of AnxA5-AH5, and/or 3.5μg/mice of AH5 peptide on days 11, 12, 14, and 15. On day 16, tumor tissues and serum of mice were harvested. **(A)** Schematic diagram. **(B)** Bar graphs depicting the abundance of CD11b+ F4/80+ macrophages and their M1 / M2 distributions in the tumor tissue following flow cytometry analysis (n=3). **(C)** Bar graphs depicting the presence of CD8+ T cells, CD4+ T cells, Treg cells, and MDSCs in the tumor tissue following flow cytometry analysis (n=3). **(D-E)** Bar graphs depicting the levels of TNF-α, IL-10 and TGF-β cytokines in the tumor tissue **(D)** and serum **(E)** of mice as measured by ELISA (n=3). The error bars indicate mean ± SD. For **B-E**, P-values were analyzed by Student's t-test. N.S. = not significant. The results are representative of one of three independent experiments. Source data are provided as a Source Data file.

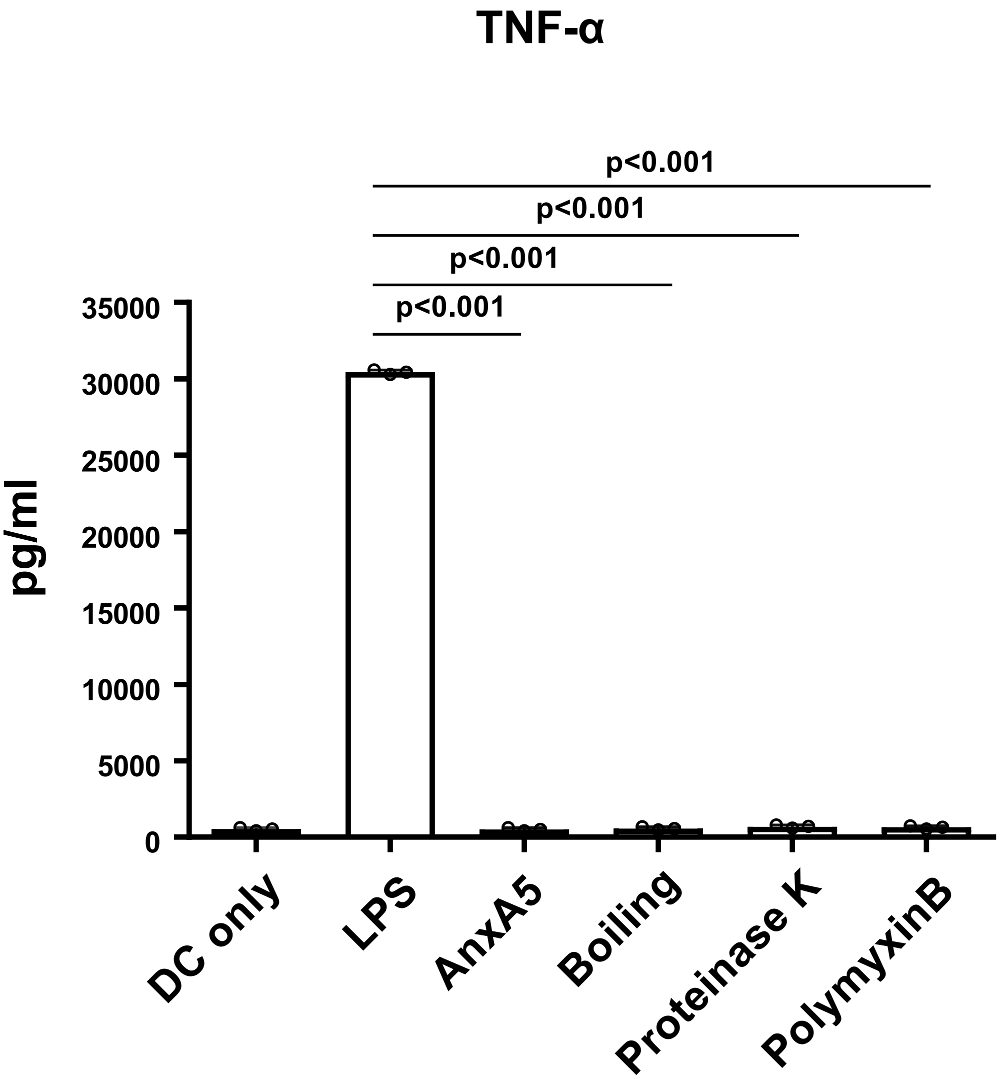

Supplementary Figure 14. Purified AnxA5 proteins are not endotoxin contaminated. DCs were treated with LPS (100ng), AnxA5 (20μg), or AnxA5 (20μg) that were boiled, treated with proteinase K (100μg) at 40°C for three hours or polymyxin B (10μg) at room temperature for ten minutes. Level of TNF-α cytokine in the supernatants of treated DCs were measured using ELISA. The error bars indicate mean ± SD. P-values were analyzed by Student's t-test. The results are representative of one of three independent experiments. Source data are provided as a Source Data file.

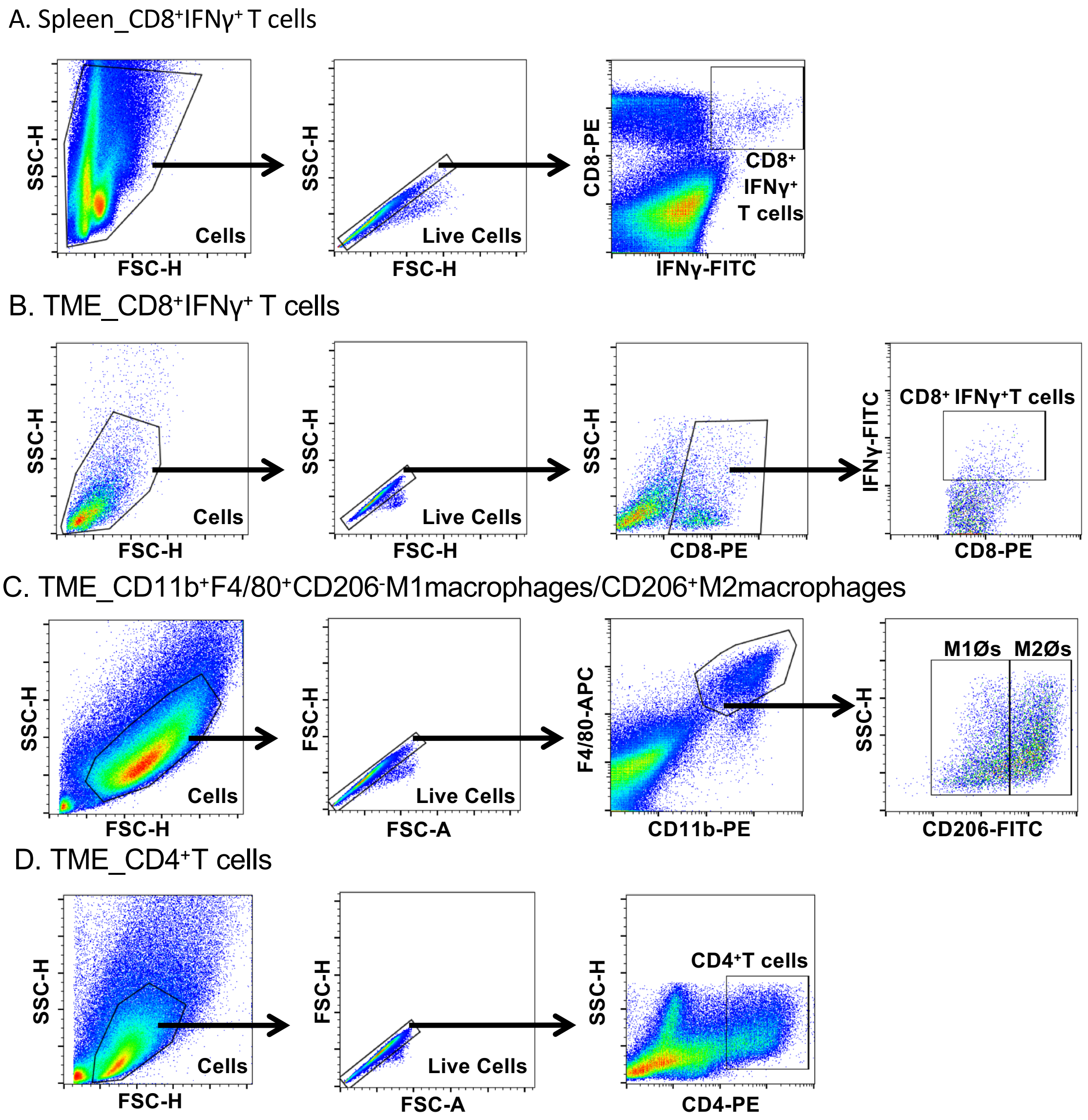

Supplementary Figure 15. Gating scheme for flow cytometric analysis of immune cell populations. Initial cell populations were gated for a live population using FSC and SSC plot of cell only sample. The gating strategy for all samples was set to remove cell debris, large clumps or aggregates of cells (large FSC and SSC) and dead cells (small FSC-H and FSC-A). The percentage reflects the ratio of specific immune cell to the total live population. A. Spleen_CD8^{+}IFNγ^{+} T cells: Figure 1D, Figure 3D, Figure 4D, Figure 6D, Figure 7D, Supplementary Figure 10D, Supplementary Figure 12D; B. TME_CD8^{+}IFNγ^{+} T cells: Figure 1E, Figure 3E Figure 4E, Figure 5E, Figure 6E, Supplementary Figure 10E; C. TME_CD11b^{+}F4/80^{+}CD206^{-}M1 macrophages /CD206^{+}M2macrophages: Figure 2B, Supplementary Figure 6B, Supplementary Figure 13B, D. TME_CD4^{+}T cells: Figure 2C, Supplementary Figure 6C, Supplementary Figure 13C

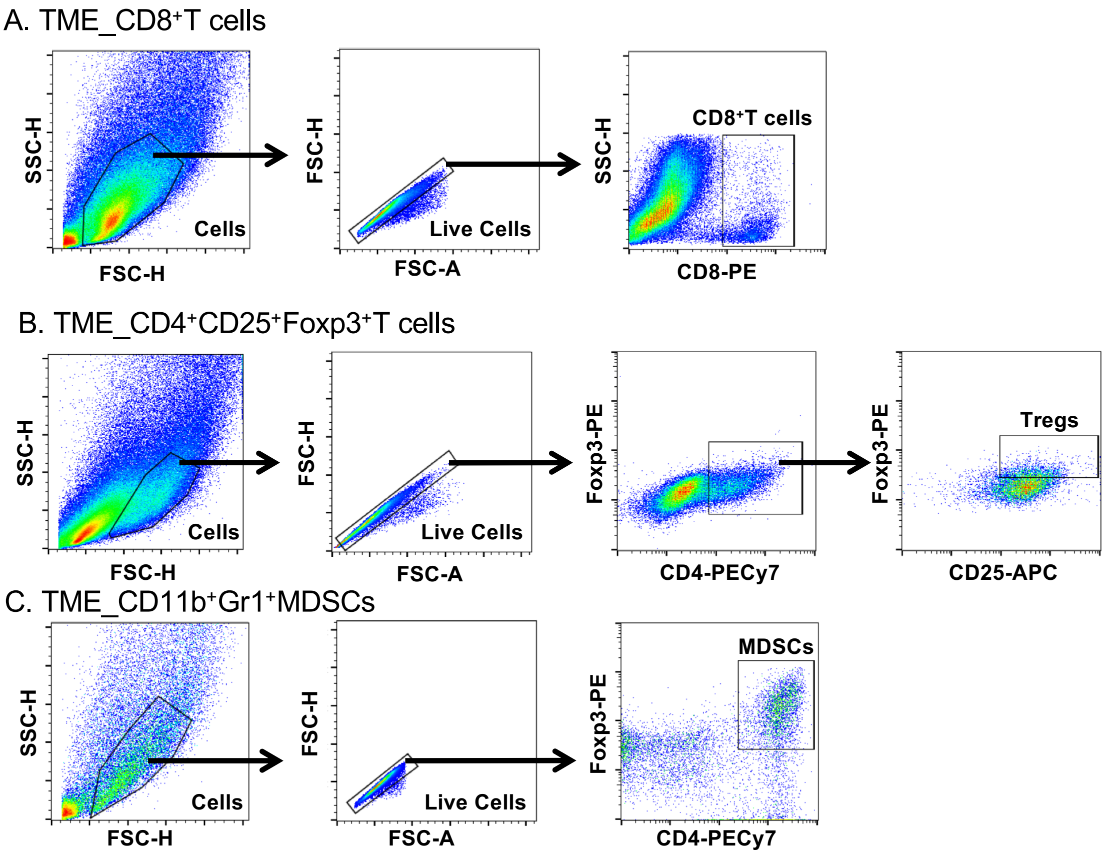

Supplementary Figure 16. Gating scheme for flow cytometric analysis of immune cell populations. Initial cell populations were gated for a live population using FSC and SSC plot of cell only sample. The gating strategy for all samples was set to remove cell debris, large clumps or aggregates of cells (large FSC and SSC) and dead cells (small FSC-H and FSC-A). The percentage reflects the ratio of specific immune cell to the total live population. A. TME_CD8^{+}T cells: Figure 2C, Supplementary Figure 6C, Supplementary Figure 13C; B. TME_CD4^{+}CD25^{+}Foxp3^{+}T cells: Figure 2C, Supplementary Figure 1C, Supplementary Figure 6C, Supplementary Figure 13C: C. TME_CD11b^{+}Gr1^{+}MDSCs: Figure 2C, Supplementary Figure 1B, Supplementary Figure 6C, Supplementary Figure 13C

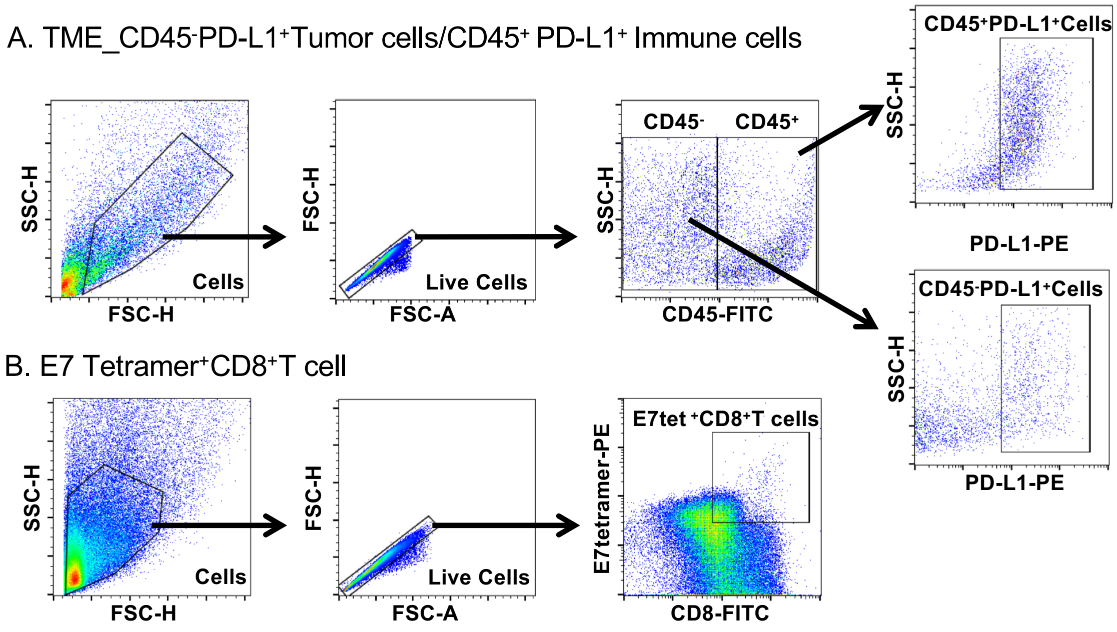

Supplementary Figure 17. Gating scheme for flow cytometric analysis of immune cell populations. Initial cell populations were gated for a live population using FSC and SSC plot of cell only sample. The gating strategy for all samples was set to remove cell debris, large clumps or aggregates of cells (large FSC and SSC) and dead cells (small FSC-H and FSC-A). The percentage reflects the ratio of specific immune cell to the total live population. A. TME_CD45$^-$PD-L1$^+$Tumor cells/CD45$^+$ PD-L1$^+$ Immune cells: Figure 2D; I. E7 Tetramer$^+$CD8$^+$T cell: Figure 5D

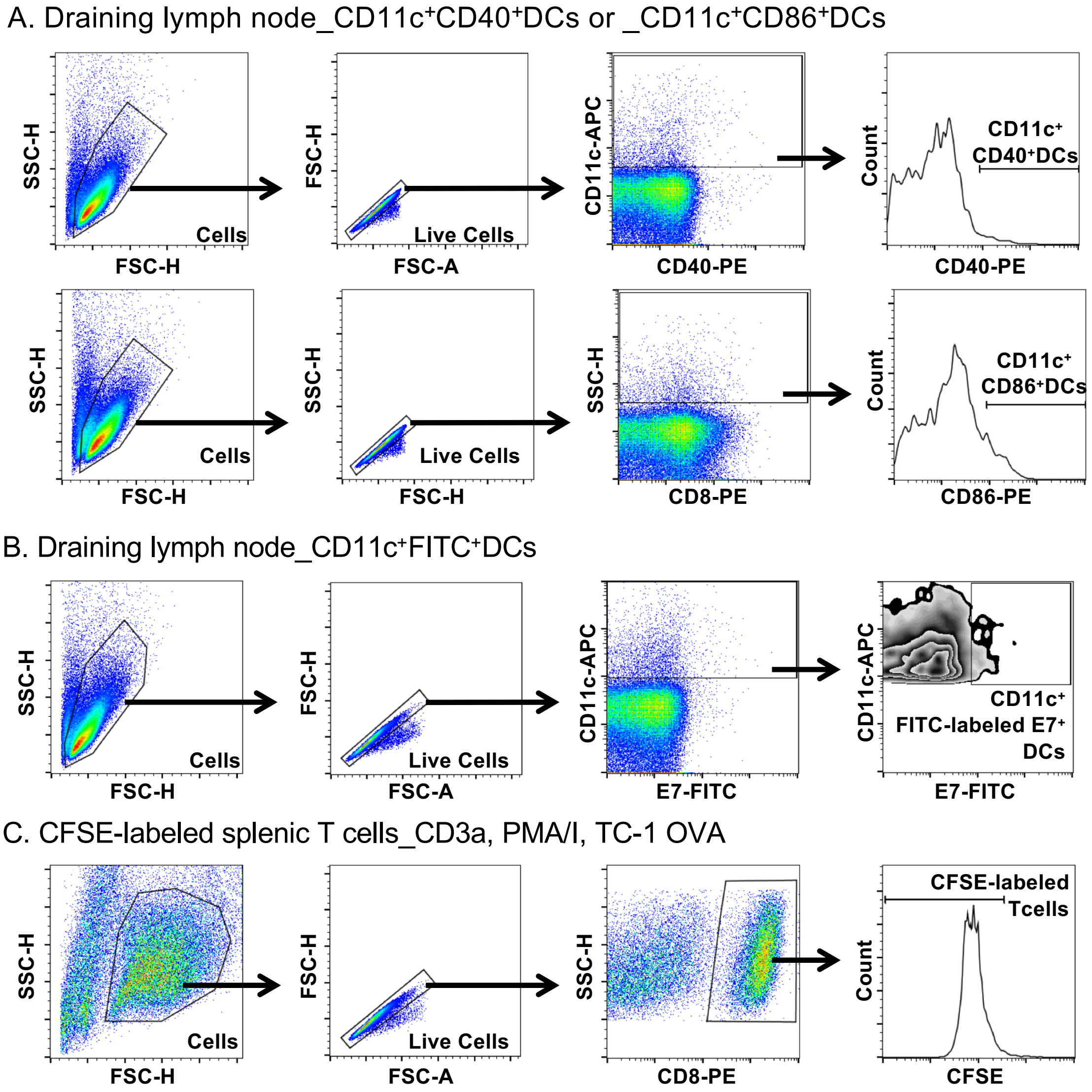

Supplementary Figure 18. Gating scheme for flow cytometric analysis of immune cell populations. Initial cell populations were gated for a live population using FSC and SSC plot of cell only sample. The gating strategy for all samples was set to remove cell debris, large clumps or aggregates of cells (large FSC and SSC) and dead cells (small FSC-H and FSC-A). The percentage reflects the ratio of specific immune cell to the total live population. A. CD11c+ CD40+ or CD86+ DCs in the draining lymph nodes: Supplementary Figure 2B; B. CD11c+ FITC+ DCs in the draining lymph node: Supplementary Figure 2C; C. T cell proliferation: Supplementary Figure 5.
